# Intestine-specific homeobox (ISX) upregulates E2F1 expression and related oncogenic activities in HCC

**DOI:** 10.18632/oncotarget.9228

**Published:** 2016-05-09

**Authors:** Shen-Nien Wang, Li-Ting Wang, Ding-Ping Sun, Chee-Yin Chai, Edward Hsi, Hsing-Tao Kuo, Kazunari K. Yokoyama, Shih-Hsien Hsu

**Affiliations:** ^1^ Division of Hepatobiliary Surgery, Department of Surgery, Faculty of Medicine, Kaohsiung Medical University Hospital, Kaohsiung, Taiwan; ^2^ Graduate Institute of Medicine, College of Medicine, Kaohsiung Medical University, Kaohsiung, Taiwan; ^3^ Division of General Surgery, Department of Surgery, Chi-Mei Medical Center, Tainan, Taiwan; ^4^ Department of Food Science and Technology, Chia Nan University of Pharmacy and Science, Tainan, Taiwan; ^5^ Department of Pathology, Faculty of Medicine, College of Medicine, Kaohsiung, Taiwan; ^6^ Department of Genome Medicine, College of Medicine, Kaohsiung Medical University, Kaohsiung, Taiwan; ^7^ Department of Internal Medicine, Division of Hepatogastroenterology, Chi-Mei Medical Center, Tainan, Taiwan; ^8^ Department of Senior Citizen Service Management, Chia Nan University of Pharmacy & Science, Tainan, Taiwan; ^9^ Research Center for Stem Cell Research, Kaohsiung Medical University, Kaohsiung, Taiwan; ^10^ Center for Environmental Medicine, Kaohsiung Medical University, Kaohsiung, Taiwan; ^11^ Department of Molecular Preventive Medicine, Graduate School of Medicine, The University of Tokyo, Tokyo, Japan; ^12^ Faculty of Science and Engineering, Tokushima Bunri University, Sanuki, Japan; ^13^ Center of Infectious Disease and Cancer Research, Kaohsiung Medical University, Kaohsiung, Taiwan

**Keywords:** cyclin D1, DP1, E2F1, hepatocellular carcinoma (HCC), ISX

## Abstract

Intestine-specific homeobox (*ISX*), a newly identified proto-oncogene, is involved in cell proliferation and progression of hepatocellular carcinoma (HCC). However, the underlying mechanisms linking gene expression and tumor formation remain unclear. In this study, we found that *ISX* transcriptionally activated E2F transcription factor 1 (*E2F1*) and associated oncogenic activity by directly binding to the E2 site of its promoter. Forced expression of *ISX* increased the expression of and phosphorylated the serine residue at position 332 of *E2F1*, which may be translocated into the nucleus to form the *E2F1*–DP-1 complex, suggesting that the promotion of oncogenic activities of the *ISX–E2F1* axis plays a critical role in hepatoma cells. Coexpression of *ISX* and *E2F1* significantly promoted p53 and RB-mediated cell proliferation and anti-apoptosis, and repressed apoptosis and autophagy. In contrast, short hairpin RNAi-mediated attenuation of *ISX* and *E2F1* decreased cell proliferation and malignant transformation, respectively, in hepatoma cells *in vitro* and *in vivo*. The mRNA expression of *E2F1* and *ISX* in 238 paired specimens from human HCC patients, and the adjacent, normal tissues exhibited a tumor-specific expression pattern which was highly correlated with disease pathogenesis, patient survival time, progression stage, and poor prognosis. Therefore, our results indicate that *E2F1* is an important downstream gene of *ISX* in hepatoma progression.

## INTRODUCTION

Intestine-specific homeobox (ISX), a newly identified proto-oncogene, regulates cell proliferation and drives hepatocellular carcinoma (HCC) formation via cyclin D1 upregulation under stimulation by proinflammatory cytokines such as IL-6 [1]. In our previous study, upregulation of E2F transcription factor 1 (E2F1) was detected in a forced ISX expression profile, but the underlying molecular signal circuits and clinical outcomes remain unclear [1]. E2F family transcription factors play key roles in cell cycle progression, apoptosis, cell differentiation, and stress response [2, 3]. Deregulated expression of the E2F family of transcription factors is a common phenomenon in human cancers [4, 5]; however, because little is known about the magnitude and nature of this deregulated expression, the relationship between oncogenes and E2F1 expression in human cancer is complex, and more extensive investigation is required.

E2F1 executes most of its biological functions through its ability to activate transcription in downstream genes involved in the cell cycle during the G_1_–S-phase transition, DNA synthesis and replication, checkpoint control, DNA damage and repair, apoptosis, autophagy, self-renewal, development, and differentiation [6, 7]. However, E2F1 also has transcription-independent activities that facilitated DNA repair or induce autophagy and apoptosis [8–10]. E2F1-knockout animals display testicular atrophy, exocrine gland dysplasia, and maturation stage defects in thymus cells apoptosis, suggesting a role of E2F1 in apoptosis [11–13]. The role of E2F1 in recruiting other transcription factors and co-factors has not been thoroughly investigated and certainly deserves more attention, which is more than likely to increase the biological complexity of E2F1. The cell cycle regulatory activity of E2F1 is controlled through the temporally regulated physical association of retinoblastoma proteins 1 (RB1), also known as “pocket” proteins, with E2F subunits, whereby tumors with a deregulated E2F1/RB1 network cannot promote p53-dependent apoptosis under conditions of p53 mutation or MDM2 overexpression [2]. E2F1 appears to play different regulatory roles in human malignancies; E2F1 shows tumor-suppressing activity in esophageal, gastric, and colorectal adenocarcinoma, whereas it may function as a tumor promoter in pancreatic ductal adenocarcinoma and esophageal squamous cell carcinoma [5, 14, 15]. In HCC, E2F1 expression has shown to be controversial in terms of the pro- and anti-apoptotic effects on tumorigenesis [16, 17]. On one hand, E2F1 acts as an inhibitor of hepatitis B virus-mediated HCC by activating p53 expression [18, 19]. In animal models, the TFDP3/E2F1 pathway induces apoptosis in HCC by positively regulating HIF-2α, and the decreased levels of HIF-2α were associated with lower overall survival of HCC animals [20, 21]. On the other hand, overexpression and genome amplification of E2F1 has been observed in HCC [17, 22]. E2F1 may counteract c-MYC-driven apoptosis via activation of the PIK3CA/AKT/mTOR, c-MYB/COX-2, and MYBL2 [23] pathways in human and rodent liver cancer [24–26]. Furthermore, E2F1 has recently been shown to be a fibrogenic protein that promotes liver fibrosis, a pre-stage of HCC, via interaction with SHP and its co-repressor EID1, to control Egr-1 expression in non-alcoholic and alcoholic liver fibrosis/cirrhosis [13].

In this study, we show that ISX transcriptionally activates E2F1 expression by directly binding to the *E2F1* promoter to activate the oncogenic activity of E2F1 via dissociation from RB1 and nuclear translocation. E2F1 plays an important role in oncogenic activities, instead of in apoptosis and autophagy in HCC progression, by coupling with the expression of upstream oncogenes, such as *ISX*. Thus, *E2F1* is a critical target gene of *ISX* in hepatoma progression.

## RESULTS

### Expression of E2F1 is upregulated by ISX in hepatoma cell lines

**Figure 1:**
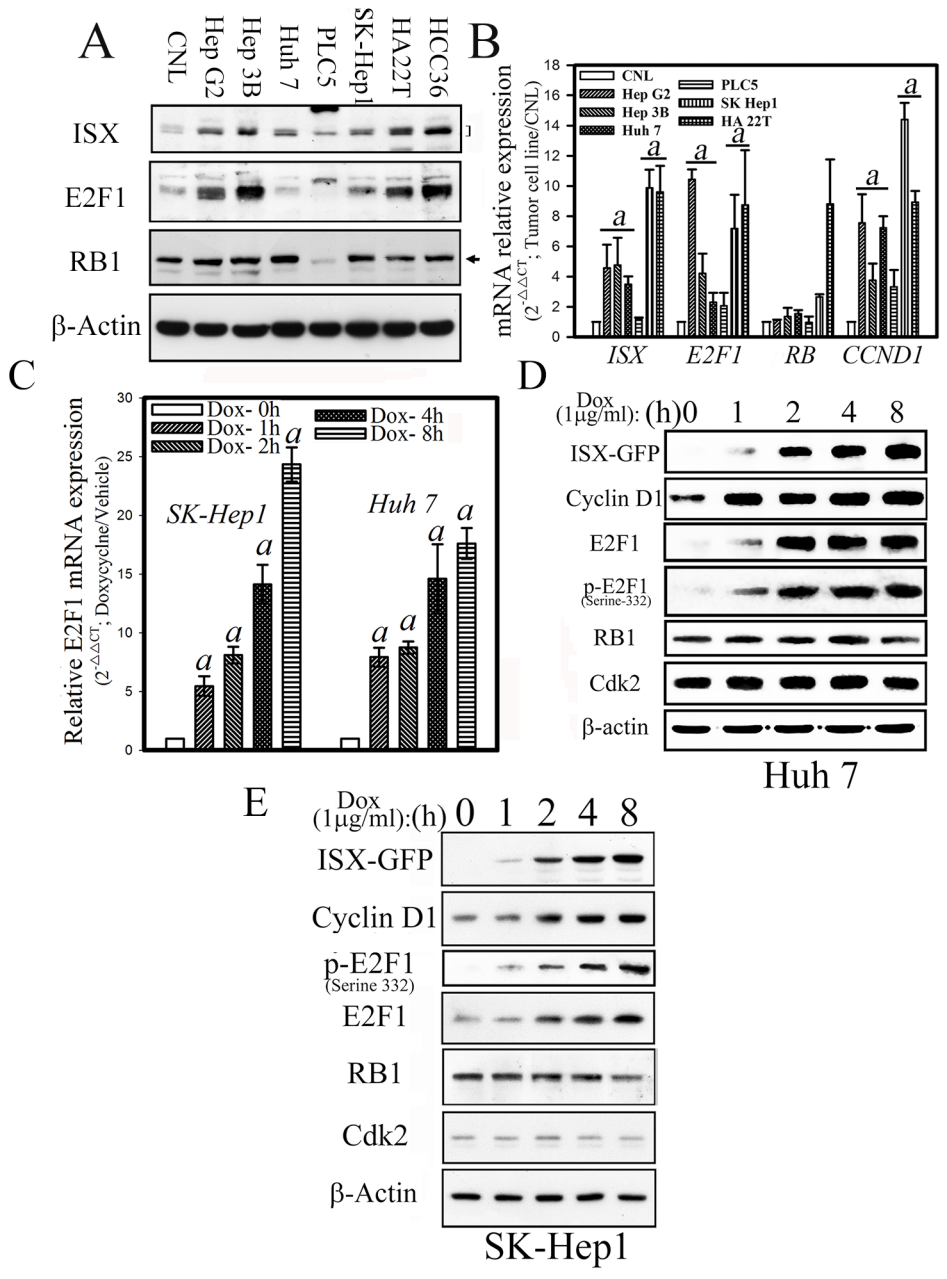
Forced ISX expression upregulates E2F1 in hepatoma cells **A.** Western blots analysis of ISX, E2F1, and RB protein expression in various hepatoma cells. CNL: Chang normal liver cells. **B.** Relative mRNA expression levels of ISX, E2F1, RB, and cyclin D1 in hepatoma cells. Data are presented as means ± S.D. a, *p* < 0.001. **C.** Time course of relative E2F1 mRNA expression in SK-Hep1 and Huh 7 cells after induction with doxycycline (1 μg/ml). **D.** Expression of cell cycle regulatory proteins in Huh 7 cells after induction of *ISX-GFP* by doxycycline (1 μg/ml). **E.** Expression of cell cycle-associated proteins in SK-Hep1 cells after induction of *ISX-GFP* with (1 μg/ml).

Analyses of seven hepatoma cells (Hep G2, Hep 3B, SK-Hep1, Huh 7, PLC/PRF/5, HA 22T, and HCC36) revealed that the mRNA and protein expression patterns of ISX and E2F1 were co-expressed significantly (3.5–9.9-fold) in hepatoma cells (Hep G2, Hep 3B, SK-Hep1, HA 22T, and HCC36) relative to those of benign hepatocytes (Chang normal liver cells, CNL; Figure 1A and 1B). In addition, in two ISX-inducible hepatoma cells (SK-Hep1 and Huh 7), the mRNA of *E2F1* and protein of total E2F1, cell cycle-associated phosphorylated E2F1 (332^serine^), and cyclin D1–a positive marker of an *ISX* downstream gene–all were shown to increase 5.6–24.8-fold in a time-dependent manner after the induction of ISX by doxycycline (Dox.; 1 μg/ml) (Figure 1C, 1D and 1E).

### ISX transactivates *E2F1* promoter through E2 *cis*-element

**Figure 2:**
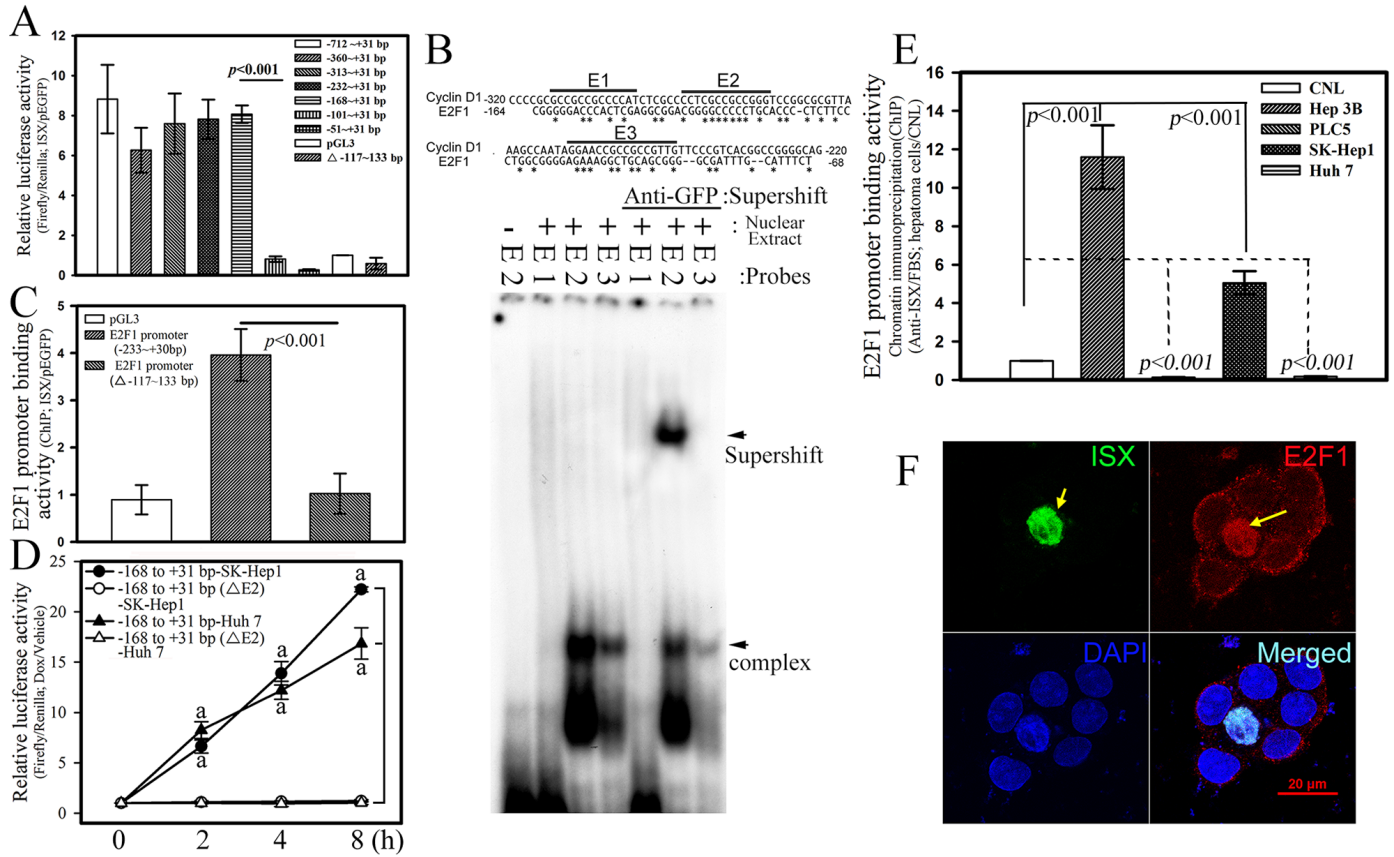
ISX transactivates *E2F1* promoter **A.** ISX transcriptionally activated luciferase activity driven by *E2F1* promoter in Hep 3B cells. Indicated deletion luciferase mutants were constructed as described in the Materials and Methods. **B.** EMSA analysis of ISX protein bonded directly to the DNA element region (−133 to −117 bp) on the E2F1 promoter *in vitro*. The preparation of nuclear extracts and EMSA assay were performed as described in the Materials and Methods. **C.** E2F1 promoter region (−233 to +31 bp) was immunoprecipitated with anti-ISX antibody using extracts from SK-Hep1 transfected cells. The DNA element region (−133 to −117 bp) on the E2F1 promoter is essential for the promoter-binding activity of ISX on *E2F1* expression *in vivo*. **D.** The transactivation activity of ISX on the E2F1 promoter was determined by luciferase assay as described in the Materials and Methods. a, *p* < 0.001. **E.** Chromatin was prepared and immunoprecipitated with anti-ISX antibody from different hepatoma cells. The DNA-binding activity of ISX on the E2F1 promoter was determined in different hepatoma cells. **F.** Forced ISX induced E2F1 expression and nuclear translocation of ISX–E2F. ISX and E2F1 were detected by immunofluorescence as described in the Materials and Methods. ISX, green; E2F1, red; and nucleus (4′,6-diamidino-2-phenylindole [DAPI]), blue (DAPI). Hep 3B cell transfected with ISX (yellow arrow). The results in A, C, D, and E are shown as means ± S.D. Each experiment was repeated three times.

A promoter assay, electrophoresis mobility shift assay (EMSA), and chromatin immunoprecipitation (ChIP) assays were used to investigate the potential regulatory effects of ISX on E2F1 expression. First, the *E2F1* promoter with serial deletions was subcloned into a luciferase expression construct to identify the potential regulatory region controlled by ISX (Figure 2A). ISX significantly increased the *E2F1* promoter-driven luciferase activity (6.2–8.8-fold) compared with that in the mock-transfected cells until the promoter sequence was shorter than −101 bp in SK-Hep1 cells (Figure 2A). From the analysis of the *E2F1* promoter region between positions −168 bp and −101 bp, three potential ISX-binding motifs (E1 to E3) were identified and synthesized for EMSA analysis *in vitro* (Figure 2B). These *cis* elements were also observed in the promoter region of *cyclin D1* [1]. Nuclear ISX proteins extracted from Hep 3B cells transfected with *pEGFP/c1-ISX* showed high affinity to the E2 motif (positions −132 to −117 bp) and the E2–ISX complex was supershifted by the addition of an anti-GFP antibody, but not supershifted with other E1 and E3 sites as probes. Hepatoma cells (SK-Hep1) that were cotransfected with deletion mutants of the *E2F1* promoter (positions delta−117 to −133) and *pEGFP/c1-ISX* lost the luciferase activity induced by ISX (Figure 2A). The comparative transactivation effect of *pEGFP/c1-ISX* on the *E2F1* promoter using positions −168–+31 and delta−117–−132 was further examined and confirmed by an *in vivo* DNA-binding assay (Figure 2C). The *E2F1* promoter regions (positions −168 to +31bp) were pulled down by the addition of anti-GFP monoclonal antibodies in SK-Hep1 hepatoma cells transfected with *pEGFP/c1-ISX* expression vector. In contrast, the E2F1 mutant with E2 motif deletion was not effective for the recruitment of ISX (Figure 2C). The transactivation effect of ISX on *E2F1* promoter was further confirmed by a luciferase assay. Hepatoma cells transfected with E2F1 mutant with E2 motif deletion showed no luciferase activity induced by ISX expression (Figure 2D). The chromatin-binding activity of ISX in four hepatoma and hepatocyte cells was analyzed by the ChIP assay. The *E2F1* promoter region between −168 and +31 was pulled down by an anti-ISX antibody and was shown to correlate with the expression level of E2F1 in hepatoma cells, particularly in Hep3B and SK-Hep1 cells (Figure 2E). These results indicate that ISX controls E2F1 expression by binding to the potential ISX binding element E2 (−132 to −117 bp) on the *E2F1* promoter sequence.

### ISX induces E2F1 nuclear translocation

**Figure 3:**
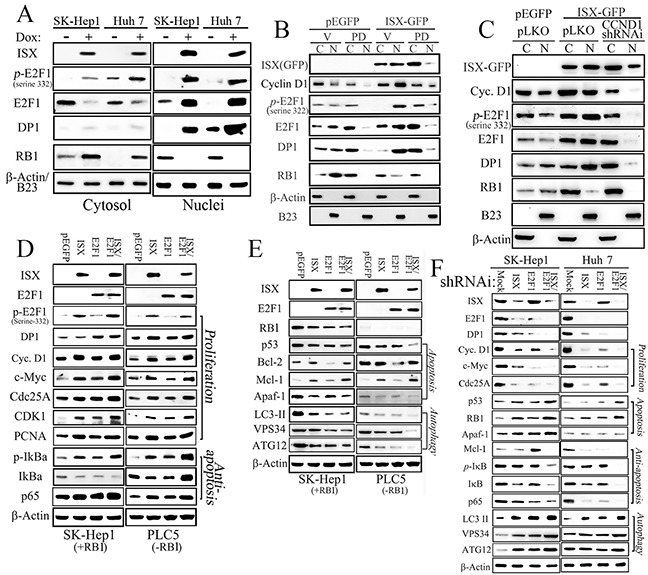
ISX enhances E2F1 expression and nuclear translocation **A.** ISX induces the expression of p-E2F1 (serine 332) and DP1 in the nucleus. **B.** A cyclin D1–CDK4/6 inhibitor, PD 0332991 (PD; 30 nM) reduced the expression of E2F1 and p-E2F1 in the nucleus in response to ISX-GFP. C, cytoplasm; N, nuclei. **C.** The hepatoma cells transfected with cyclin D1-specific shRNAi decreased the expressions of p-E2F1, E2F1, DP-1, and RB1 in the nucleus in response to *ISX-GFP*. **D.** Expressions of various proliferation and anti-apoptosis related proteins in cells [SK-Hep1 (RB1^+^) and PLC5 (RB1^−^)] transfected with ISX and/or E2F1 genes. **E.** Forced coexpression of ISX and E2F1 inhibits apoptotic and autophagic signaling in SK-Hep1 (RB1^+^) and PLC5 (RB1^−^) cells. **F.** Hepatoma cells (SK-Hep1 and Huh 7) cotransfected with ISX and E2F1 shRNAi showed dramatically decreased proliferation and anti-apoptotic signals but increased apoptotic and autophagic signals from those in the cells transfected with *ISX* or *E2F1* shRNAi alone. Assays were performed three times.

Besides upregulation of E2F1 expression, the potential cellular effects of E2F1 induced by ISX were further determined by immunofluorescent staining in Hep 3B cells transfected with *pEGFP/c1-ISX* (Figure 2F; confocal images). Upregulated and nuclear-localized E2F1 (red), as the overexpressed ISX protein (green), was detected mainly in the nucleus (blue) of Hep 3B cells with forced ISX expression (yellow arrow) rather than in neighbor hepatoma cells without overexpressed ISX. The expression pattern of E2F1 protein induced by ISX was further investigated in SK-Hep1 and Huh 7 cells, which have lower expression of ISX, and after the forced expression of ISX, a significantly enhanced E2F1 protein was detected in the cytoplasm and nucleus, although the nuclear E2F1 showed 3- or 5-fold higher expression than that in the cytoplasm (Figure 3A). The phosphorylated E2F1 at position 332 serine residue by Cyclin D1-CDK4/6 [12, 18] was induced significantly (more than 10–20-fold) and localized in the nucleus in ISX overexpressed SK-Hep1and Huh 7 cells (Figures 1D, 1E and 3A).

Similar to E2F1, DP1 was also shown to be upregulated more than 10-fold in the nucleus after the induction of ISX (Figure 3A). In contrast, RB1 in hepatoma cells with ISX overexpression after Dox induction was shown to translocate into the cytoplasm in SK-Hep1 and Huh 7 cells. SK-Hep1 cells treated with PD 0332991(IC50 = 10 nM), a highly selective inhibitor of cyclin D1–CDK4/6, or transfected with cyclin D1-specific shRNAi (84% knockdown efficiency) were shown to block (by 62% and 78%, respectively) the translocation of phosphorylated E2F1 (serine 332) to the nucleus (Figure 3B and 3C).

### Effect of *ISX* on cell proliferation, anti-apoptosis, apoptosis, and autophagy activities in HCC cells

To characterize the cellular functions of E2F1, RB1, and ISX, wild type ISX tagged with GFP and wild type E2F1 tagged with HA were transfected into SK-Hep1 (with higher expression of RB1) and PLC5 (with no RB1 expression) cells. Forced expression of ISX in hepatoma cells (SK-Hep1 and PLC5) significantly increased the expression of proliferation markers (e.g., Cyclin D1, c-Myc, Cdc25A, and PCNA), anti-apoptosis markers (p65 signal related proteins, Bcl-2, and Mcl-1), cell cycle markers like E2F1, and phospho-E2F1 (p-E2F1; serine 332) but not DP1 protein (Figures 3D, 3E and S1A). Similarly, forced expression of E2F1 enhanced the expression of some proliferation markers (e.g., c-Myc, Cdc25A, and CDK1, with less effect on PCNA) and anti-apoptotic markers (e.g., p65 signals). Forced coexpression of both ISX and E2F1 genes upregulated DP1 expression, but it seems to be an additive effect on most proliferation and anti-apoptotic markers (Figures 3D, 3E and S1A).

By contrast, forced expression of ISX reduced the expression of apoptotic (RB1, p53, and Apaf1) and autophagic markers (VSP34, ATG12, and LC-3II), and forced expression of E2F1 alone downregulated anti-apoptotic (Bcl-2) and autophagic markers (VSP34, ATG12, and LC-3 II) (Figures 3E and S1B). Accordingly, forced coexpression of both *ISX* and *E2F1* genes repressed the expressions of tumor suppressor protein p53, apoptotic (Apaf-1), and autophagic markers (VSP34, ATG12, and LC-3II) in both SK-Hep1 and PLC5 cells (Figures 3E and S1B).

The cellular function of the ISX–E2F1 axis was evaluated by gene-specific short hairpin RNA interference (shRNAi) in hepatoma cells. Hepatoma cells (SK-Hep1 and Huh 7) transfected with *ISX* shRNAi showed significant downregulation of E2F1 and DP1 protein expression as well as of proliferation markers (cyclin D1, c-Myc, Cdc25A, and PCNA) and anti-apoptotic genes (p65 and Mcl-1) (Figures 3F and S1C). However, the tumor suppressor gene products (p53 and RB1) and apoptotic (Apaf-1) and autophagic markers (VSP34, ATG12, and LC-3II) increased 2–6.2-fold in hepatoma cells transfected with *ISX* shRNAi (Figures 3E and S1C). As with *ISX* shRNAi, hepatoma cells transfected with *E2F1* shRNAi showed decreased expression of DP1 as well as of a proliferation and anti-apoptotic marker (p65), but increased expression of RB1, p53, Apaf-1, Mcl-1, VSP34, ATG12, and LC-3II. Hepatoma cells co-transfected with shRNA is against both *ISX* and *E2F1* showed more significant downregulation of proliferation- and anti-apoptotic signaling markers, but increased expression of apoptotic and autophagic signaling markers (Figures 3F and S1C). These results indicate that the coexpression of an oncogene (*ISX*) and *E2F1* results in oncogenic activity in SK-Hep1 and Huh 7 cells that clearly promotes tumor progression.

### Forced coexpression of *ISX* and *E2F1* abolishes E2F1-mediated apoptotic and autophagy activities

**Figure 4:**
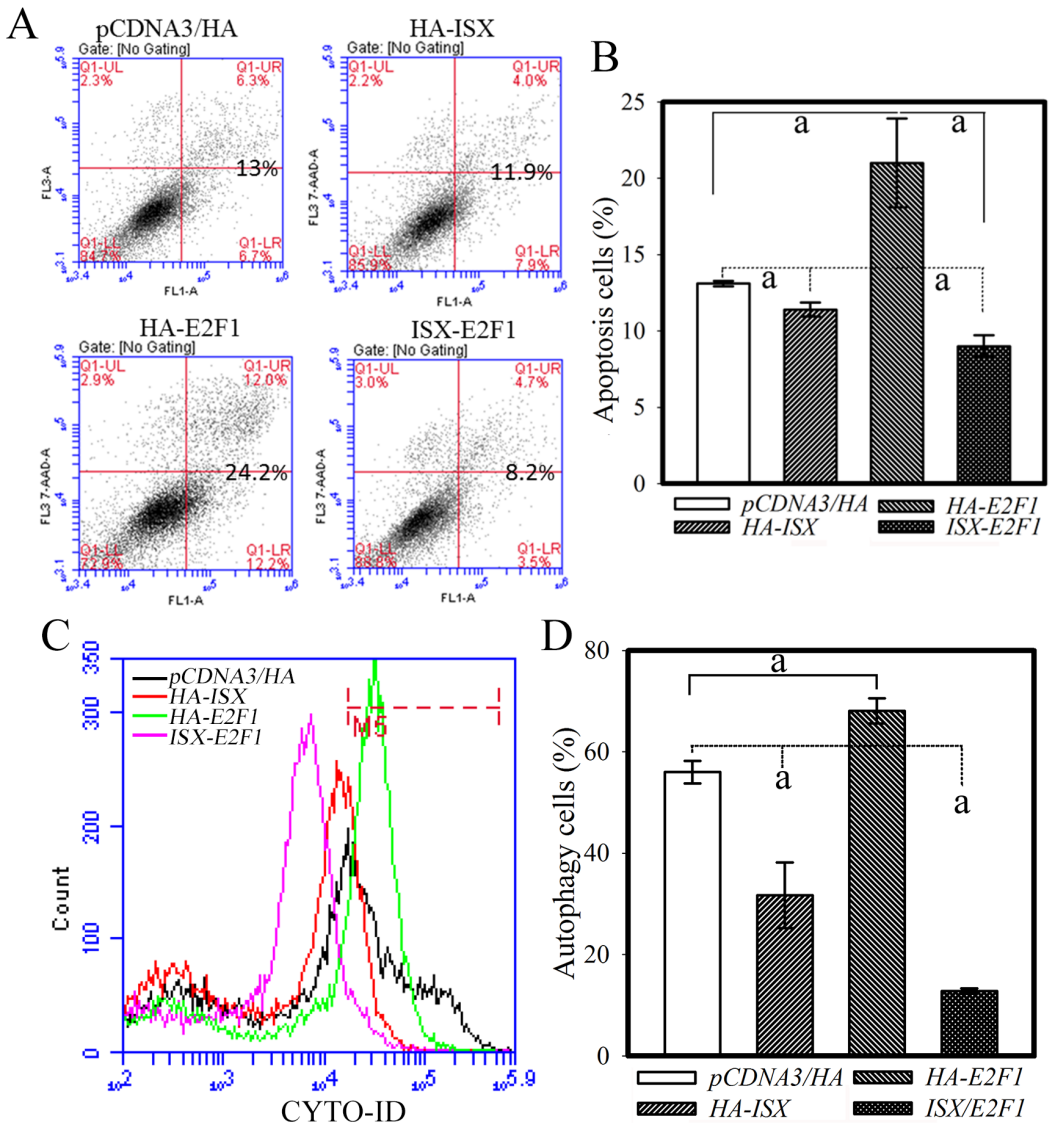
Coexpression of *ISX* and *E2F1* decreases apoptotic and autophagic activities in Huh 7 cells A. Hepatoma cells transfected with *ISX* or co-transfected with *ISX* and *E2F1* showed significantly reduced tamoxifen-induced apoptotic activity. The cells were co-stained with annexin V-FITC/PI and apoptotic cells were measured as described in the Materials and Methods. B. Statistical analysis of apoptotic cells from hepatoma cells with forced ISX and/or E2F1 expression. a, *p* < 0.001. C. Huh 7 cells transfected with *ISX* or co-transfected with *ISX* and *E2F1* showed decreased autophagic cells induced by tamoxifen treatment (30 μM) for 8 h. The autophagy cells were incubated with Cyto-ID green fluorescent probes to detect autophagic vacuoles and analyzed by flow cytometry as described in the Materials and Methods. D. Statistical analysis of autophagic cells from a population of hepatoma cells with forced ISX and/or E2F1 expression treated with tamoxifen for 8 h. a, *p* < 0.001.

The cellular effects of the ISX-E2F1 axis on apoptotic and autophagy activities were further investigated in hepatoma cells (Huh 7) with forced expression of *ISX* and *E2F1*. Huh 7 cells with forced expression of *ISX* alone or coexpression of *ISX* and *E2F1* showed significant reductions (16% and 65%, respectively) in apoptotic cells compared with those in the Huh 7 cells transfected with only the vector after treatment with tamoxifen (30 μM) for 8 h (Figure 4A and 4B). Huh 7 cells with forced expression of *E2F1* alone showed significant increases in apoptotic cells (65%) compared with those in the Huh 7 cells transfected with vector only after treatment with tamoxifen (30 μM) (Figure 4A and 4B). Also, Huh 7 cells with forced expression of *ISX* alone or coexpression of *ISX* and *E2F1* showed dramatic reductions in autophagic cells (44 and 70%, respectively) from those of the Huh 7 cells transfected with vector only after treatment with tamoxifen (30 μM) for 8 h (Figure 4C and 4D). Huh 7 cells with forced expression of *E2F1* alone showed significantly increased autophagic cells (21%) from those of the Huh 7 cells transfected with vector only after treatment with tamoxifen (30 μM) (Figure 4C and 4D). Thus, coexpression of *ISX* and *E2F1* abolished E2F1-mediated apoptosis and autophagy activity.

### E2F1 and ISX control cell proliferation and transformation activities

**Figure 5:**
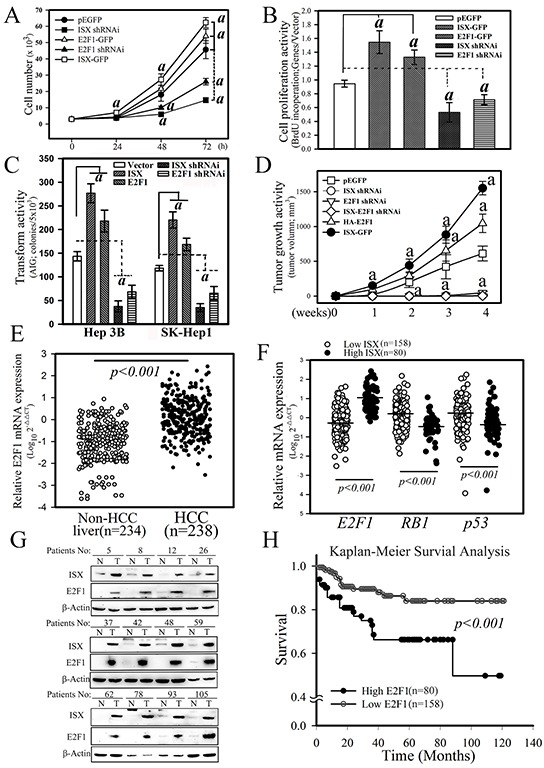
ISX enhances E2F1-mediated cell proliferation and oncogenic activity A. Effect of ISX and E2F1 on the cell growth of SK-Hep1 cells transfected with forced expression vectors of *ISX-GFP* and/or *E2F1-GFP* and knockdown vector of *ISX* and *E2F1*. SK-Hep1 cells expressed endogenous ISX. a, p < 0.001. B. BrdU incorporation of SK-Hep1 cells transfected with *ISX-GFP* and/or *E2F1-GFP* and knockdown vector of *ISX* and *E2F1*. a, p < 0.0001. C. Cell anchorage-dependent transformation activity detected by soft agar colony formation. Hep 3B and SK-Hep 1 cells were transfected with forced expression vector of *ISX* and *E2F1* and knockdown vector shRNAi against *E2F1* and *ISX*. a, p < 0.001. D. Tumor growth activity of SK-Hep1 cells transfected with forced expression vector of *ISX* and *E2F1* and knockdown vector shRNAi against *E2F1* and *ISX*. a, p < 0.001. E. Comparison of the mRNA expression of *E2F1* between HCC tumor and non-tumor tissues as described in the Materials and Methods. F. The mRNA level of *E2F1* in HCC patients with high ISX expression was significantly higher than that with the low *ISX*-expressing HCC patients. Both *RB1* and *p53* mRNA expressions in the high *ISX* expression HCC group were significantly downregulated relative to those in the low ISX expression HCC group. G. Western blot of ISX and E2F1 proteins in HCC patient tissues. A high protein level of E2F1 was observed in HCC patients with higher ISX. *H.* The Kaplan–Meier survival curve analysis between HCC patients with low and high expression of *E2F1*. All results are shown as means ± S.D. a, p < 0.001. Each experiment was repeated three times.

The cellular and oncogenic activities regulated by the ISX–E2F1 axis were further determined in terms of their proliferation, transformation, and tumor growth activity *in vitro* and *in vivo*. First, SK-Hep1 hepatoma cells with forced expression of *ISX* or *E2F1* showed increased cell proliferation activity from that in mock-transfected cells as assessed by cell counting at 72 h after cultivation (1.4- and 1.2-fold, respectively) and a bromodeoxyuridine (BrdU) incorporation assay (1.6-fold and 1.4-fold, respectively) (Figure 5A and 5B). Hepatoma cells treated with *ISX* or *E2F1* shRNAi showed significant decreases in growth rate (69% and 43%, respectively, according to cell counts at 72 h cultivation; 44% and 25%, respectively, according to the BrdU incorporation assay) from those observed in cells with pEGFP controls (Figure 5A and 5B). SK-Hep1 cells with overexpressed *E2F1* or *ISX* showed increased transformation (by 42% and 86%, respectively) and oncogenic activity (by 71% and 153%, respectively), whereas *E2F1* or *ISX*-knockdown SK-Hep1 cells displayed decreased transformation (by 44% and 70%, respectively) and oncogenic activities (by 92% and 98%, respectively), as determined by soft agar anchorage-independent foci formation *in vitro* and tumor growth in nude mice *in vivo* (Figure 5C and 5D). These results suggested that E2F1 regulates oncogenic activity and tumor growth induced by ISX in hepatoma cells.

### Patient characterization and clinical correlates of ISX-E2F1 axis

The experiments described above suggest that increased expression of E2F1 induced by ISX may promote cell proliferation and transformation in hepatoma cells. To verify the pathological activity and to explore the associated clinical outcomes of the ISX–E2F1 axis in HCC, 238 HCC patients with adequate follow-up data for analysis were enrolled in an ISX–E2F1 cohort study. *E2F1* mRNA expression in the HCC samples was significantly upregulated relative to that in the non-HCC liver samples (Figure 5E; *p* < 0.0001, *Mann–Whitney*). Analysis of the dichotomized group according to the level of *E2F1* expression indicated that the top 30^th^ percentile (*N* = 80; “high *E2F1*”) showed distinct clinical features from the rest of the HCC patients (*N* = 158; “low *E2F1*”). Comparison of the two groups with non-HCC subjects showed no differences with respect to age, sex, or serum levels of alanine aminotransferase, aspartate aminotransferase, bilirubin, α-fetoprotein, AC sugar, and cholesterol (Table 1). The comparison of the two HCC group subjects showed significant differences in tumor grade (*p* = 0.0071) and lymphovascular invasion activities (*p* = 0.0236) (Table 2).

**Table 1:** Baseline characteristics of 238 hepatocellular carcinoma (HCC) patents and 153 non-HCC patients

Group	Non-HCC (n=153) (n(%))	*E2F1* mRNA (Low, n=158) (n(%))	*E2F1* mRNA (High; n=80) (n(%))	*p-value*
Age (mean(SD))	61.6±5.04	61.7±0.9	56.7±1.7	0.0045
Sex				0.4976
Male	97(63.40)	123(77.85)	60(75.00)	
Female	56(36.60)	35(22.15)	20(25.00)	
GOT (U/L)				0.8003
<40	135(88.24)	72(45.60)	33(41.25)	
40 ≤ <100	10(6.53)	70(44.30)	37(46.25)	
100≤	8(5.23)	16(10.10)	10(12.50)	
GPT (U/L)				0.7978
<40	136(88.89)	65(41.14)	32(40.00)	
40≤<100	9(5.88)	78(49.37)	38(47.50)	
100≤	8(5.23)	15(9.49)	10(12.50)	
Albumin (mg/dL)				0.3760
<4.5	138(90.20)	133(84.18)	71(88.75)	
≥4.5	15(9.80)	25(15.82)	9(11.25)	
α-Fetoprotein (ng/mL)				0.6545
<20	137(89.54)	90(56.96)	48(60.00)	
≥20	16(10.46)	68(43.04)	32(40.00)	
Bilit				0.7487
1.5<	83(54.25)	132(83.54)	66(82.50)	
≥1.5	70(45.75)	26(16.46)	14(17.50)	
AC sugar				0.6847
<100	89(58.17)	37(23.42)	23(28.75)	
100≤ <120	42(27.45)	63(39.87)	26 (32.50)	
120≤	22(44.38)	58(36.71)	31 (38.75)	
ALP				0.5317
<40	90(58.82)	4(2.53)	2(2.50)	
40≤<100	40(26.14)	116(73.42)	53(66.25)	
100≤	23(15.03)	38(24.05)	25(31.25)	

#p values were calculated by Fisher exact test; *, p<0.05.

High E2F1 mRNA expression patients: E2F1 mRNA expression in tumor part were higher three point five folds (>3.5 folds) than adjacent normal part liver tissue.

Low E2F1 mRNA expression patients: E2F1 mRNA expression in tumor part were lesser three folds (<3.5 folds) than adjacent normal part liver tissue. (cut point by survival ROC curve)

Patients:238HCC patients from Chung Ho Memorial Hospital (238 HCC) were enrolled into the E2F1 cohort study from July 2007 to July 2014.

**Table 2:** Baseline characteristics of 238 hepatocellular carcinoma (HCC) patients and 153 non-HCC patients

Group	*E2F1* mRNA (Low, n=158) (n(%))	*E2F1* mRNA (High; n=80) (n(%))	*p-value*
Liver capsule invasion			0.4038
No	99(62.66)	45(56.25)	
Yes	59(37.34)	35(43.75)	
Lymphovascular invasion			0.0236*
No	114(72.15)	46(57.50)	
Yes	44(27.85)	34(42.50)	
Fibrosis			0.3336
Low	31(19.62)	18(22.50)	
Middle	89(56.33)	42(52.50)	
High	38(24.05)	20(25.00)	
Inflammatory activity			0.0550
Low	81(51.27)	35(43.75)	
High	77(48.73)	45(56.25)	
Size(cm)			0.0555
<2.5	55(34.81)	18(22.50)	
2.5≤	103(65.19)	62(77.50)	
Number of tumors			0.0793
1	124(78.48)	54(67.50)	
1<	34(21.52)	26(32.50)	
Modified TNM			0.0071*
I	100(63.29)	36(45.00)	
II	45(28.48)	28(35.00)	
III(IIIA and IIIB)	13(8.23)	16(20.00)	

High E2F1 mRNA expression patients: E2F1 mRNA expression in tumor part were higher three point five folds (>3.5 folds) than adjacent normal part liver tissue.

Low SPZ1 mRNA expression patients: E2F1 mRNA expression in tumor part were lesser three folds (<3.5 folds) than adjacent normal part liver tissue. (cut point by survival ROC curve)

Patients:238HCC patients from Chung Ho Memorial Hospital were enrolled into the E2F1 cohort study from July 2007 to July 2014.

There were significantly higher rates of increased *E2F1* expression in the HCC samples obtained from patients in the high *ISX* expression group than in the low *ISX* expression group (*p* < 0.001, Mann–Whitney test) (Figure 5F). Further, *RB1* and *p53* mRNA expression were significantly downregulated in the high-*ISX* expression group relative to that in the low *ISX* expression group (*p* < 0.001, *Mann–Whitney* test). The expression correlation for the mRNA of *ISX* and *E2F1* was determined in advance from protein expression levels in non- and HCC liver tissues. Twelve paired HCC and non-HCC liver tissues selected randomly from high mRNA correlation patients were blotted with respective ISX- and E2F1-specific antibodies, and a high expression correlation between ISX and E2F1 protein levels was detected (Figure 5G). To evaluate the potential prognostic value of *E2F1* expression, we analyzed the survival curves of HCC patients. The mean overall survival duration of all the patients was 120 months after tumor resection. As observed in the high *ISX* expression group, patients in the high *E2F1* group survived for a significantly shorter length of time after surgical resection than those in the low *E2F1* group (Figure 5H, *p* < 0.001).

**Figure 6:**
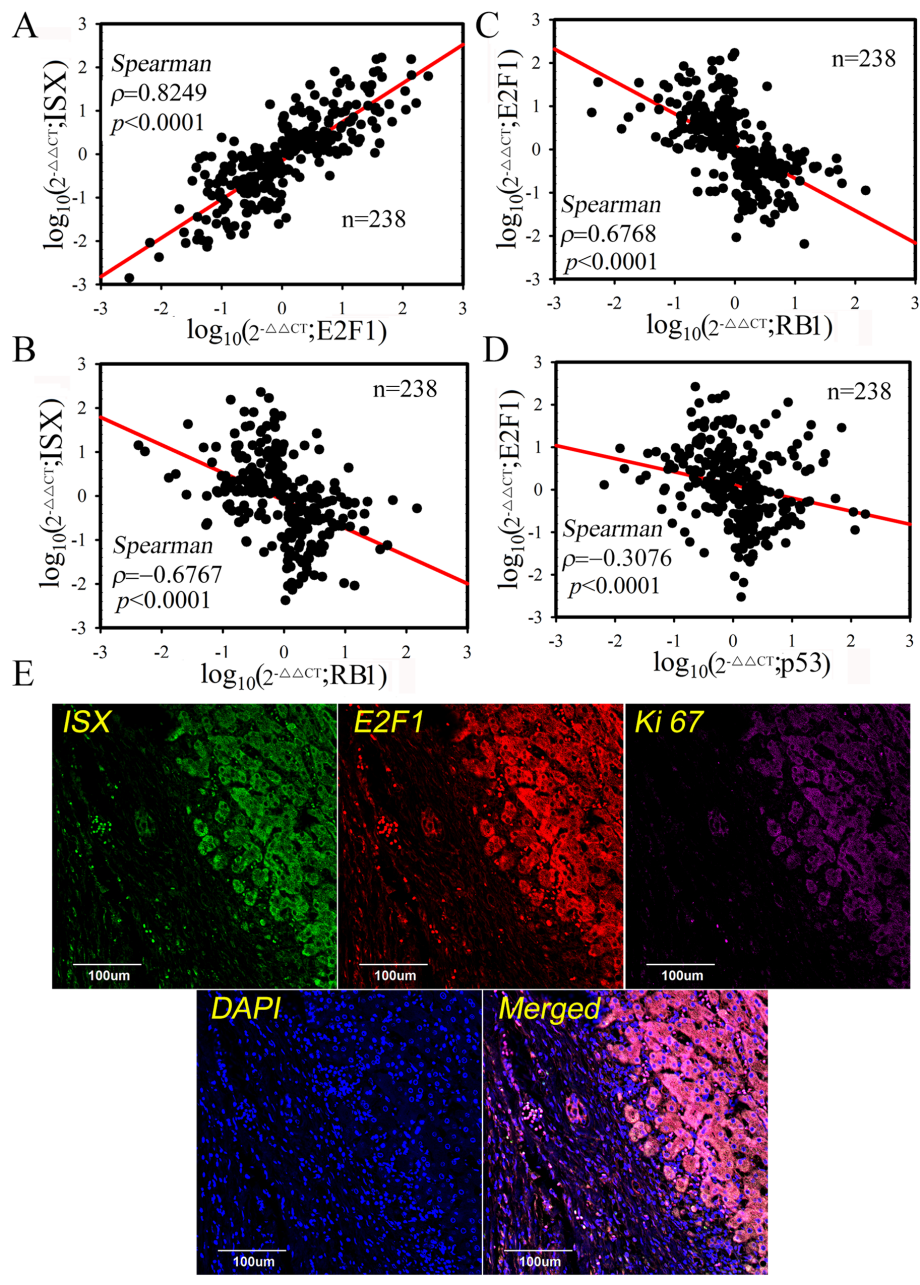
E2F1 expression was highly correlated with ISX expression in HCC patients **A, B, C,** and **D.** Spearman's correlation analysis of *E2F1*, *ISX*, *RBH,* and *p53* mRNA expression. *E2F1* mRNA expression was highly and positively correlated with *ISX* expression, but negatively correlated with *RB1* expression. **E.** ISX, E2F1, and Ki-67 were detected in HCC tumor cells by immunofluorescence staining. E2F1 proteins showed a colocalization expression pattern with ISX in the tumor cells of HCC patients. E2F1, red; ISX, green; Ki-67, pink; and nuclei, blue (4′,6-diamidino-2-phenylindole). Each experiment was repeated at least three times.

To explore the relationships among ISX, E2F1, and RB1 in non- and HCC tumors, the expression patterns of *ISX*, *E2F1*, and *RB1* mRNA were examined in non-tumor and tumor samples from 238 HCC patients. *E2F1* mRNA expression was strongly correlated with *ISX* expression in HCC patients (Spearman rank correlation coefficient; *ρ*= 0.8249, *p* < 0.0001; Figure 6A). In contrast, *RB1* mRNA expression was negatively correlated with *ISX* and *E2F1* expression in HCC patients (*ρ*= −0.6768 and −0.6767, respectively, *p* < 0.001; Figure 6B and 6C). The expression level of *E2F1* mRNA showed a strong negative correlation with the expression level of *p53* mRNA, even with lower negative coefficient (Figure 6D). This correlation between ISX and E2F1 expression was verified by confocal imaging with immunofluorescence staining of the HCC samples. E2F1 (red) and ISX (green) were more strongly expressed and colocalized in tumor masses than those in adjacent healthy liver tissues in samples obtained from HCC patients (Figure 6E). These results strongly suggest that the ISX–E2F1 axis plays a crucial role in HCC progression and is associated with patient prognosis.

## DISCUSSION

Our findings indicate that ISX is an important activator of E2F1 expression in HCC development. Mechanistically, ISX transcriptionally activates the E2F1 promoter (Figure 2A to 2D) and phosphorylates the E2F1 protein at a serine residue at position 332 (Figure 1D and 1E), probably via the cyclin D1–CDK4/6 complex [1]. It is well known that the phosphorylation of E2F1 at serine residue of position 332 leads E2F1 to dissociate from RB [12, 18] and be translocated into the nuclei to activate downstream cell cycle regulators by coupling with DP1 [1, 12, 18]. Clinical analysis also showed a significant correlation of mRNA and protein levels between E2F1 and ISX; however, both *ISX* and *E2F1* mRNA expressions were negatively correlated with RB1 expression in the HCC patients, a finding that highlights the positive cell cycle regulation and oncogenic activity of the ISX–E2F1 axis in HCC (Figure 6B, Tables 1 and 2). E2F1 regulates the expression of genes that are essential for cell proliferation but also trigger apoptosis [27]. The RB1–E2F1 axis is a major regulatory node of the cellular function of E2F1 and cell fate [28], and RB1 dissociation (or inactivation) and DP1 coupling by nuclear E2F1 are major determinants of E2F1 oncogenic activity [29]. However, the details of the underlying mechanism of E2F1 expression and associated oncogenic activation in HCC remain unclear despite the observation of significantly overexpressed E2F1 in HCC.

Here we show that ISX acts as a key regulator because ISX transactivates the *E2F1* promoter and *cyclin D1* promoter directly (Figure 2A, 2B and 2D), and ISX induces phosphorylation of E2F at the serine 332 residue, possibly leading to dissociation of the RB1–E2F1 complex in the cytoplasm [2, 30]. In nuclei, phosphorylated E2F1(serine 332) recruits DP1 to activate the expression of downstream genes involved in the G1–S-phase of cell cycle transition, initiation of DNA synthesis, and mitosis [31, 32]. These mechanistically regulatory effects of ISX on E2F1 expression and activation in HCC highlight the importance of the oncogenic activity performed by ISX in HCC. Besides the upregulation of the E2F1–DP1 complex and cell cycle regulators, the downregulation of the tumor suppressors p53 and RB1 by the ISX–E2F1 axis in hepatoma cells and tumors further emphasized the tumorigenic activity of the ISX–E2F1 axis in HCC. However, the regulatory mechanism of the expression of the tumor suppressors awaits further investigation. The shRNAi against *cyclin D1* or an inhibitor of cyclin D1 inhibited the expressions of all cell cycle regulators, E2F1, DP1, RB1, and p-E2F1 (serine 332) (Figure 3B and 3C). Given that ISX transactivates *cyclin D1* and the *E2F1* promoter (Figure 2A, 2B, and 2D), highly expressed cyclin D1 might also affect the expression of nuclear RB1, DP1, E2F1, and p-E2F1 via control of chromosomal stability [33] or of phosphorylation of E2F1, RB or DP1 via cdk4 /cdk6 kinases [34]. These positive circuit pathways should be investigated further.

E2F1 also regulated apoptosis [35, 36], senescence, and autophagy [10] under specific conditions, including DNA damage or repair [37, 38], which are correlated with tumor progression [17]. In the liver, apoptotic activity induced by forced E2F1 expression alone [16] or HBX expression [39] also showed the apoptotic role of E2F1 in hepatoma tumorigenesis [17]. In hepatoma cells with lower endogenous ISX expression, forced E2F1 alone did not upregulate apoptotic markers significantly (Figure 3E); however, a significant increase in apoptosis and autophagy activities was detected when hepatoma cells were exposed to apoptotic or autophagy stress induced by tamoxifen treatment (Figure 4). These results showed that hepatoma cells with forced coexpression of ISX and E2F1 favored proliferation and anti-apoptotic effects instead of apoptosis and autophagy (Figure 4). Thus, we propose ISX as the driver of E2F1-dependent effects.

The tumor suppressors p53 and RB1 are major regulators of cell apoptosis, senescence, and autophagy that have been shown to be induced by E2F1 expression alone in other types of human malignancies [10, 32]. Apoptotic and autophagic signaling were significantly downregulated in hepatoma cells co-transfected with ISX and E2F1 relative to that in the cells transfected with either ISX or E2F1 (Figure 4). The suppression effect of the ISX–E2F1 axis on apoptosis and autophagy in the hepatoma cells showed that the ISX–E2F1 axis favors the creation of a tumor-promoting signal for malignancies; in the tumor-promoting signals induced by ISX, the E2F1–DP1 complex transcriptionally activated cell cycle regulators instead of apoptotic or autophagic factors. The downregulation of the expression of tumor suppressors, p53 and RB1, in hepatoma cells with overexpressed ISX–E2F1 is one of the major factors in reversing the cellular response. Overexpressed cyclin D1 induced by ISX leads RB1 to hyperphosphorylation, resulting in degradation of RB1 and possibly reversing the significant reduction of nuclear RB1. These results shed light on the detailed mechanism of the tumorigenic activity and signals induced by the ISX–E2F1 axis.

E2F1 is an important oncogenic mediator in ISX-induced tumorigenesis. ISX transcriptionally activated E2F1 expression and, through cyclin D1 activation, E2F1 dissociated from RB1 and translocated into the nucleus to activate cell cycle regulators from the G1–S-phases. The results of this study emphasize the importance of the ISX–E2F1 axis, an oncogenic transcription factor, in HCC generation via the creation of an oncogenic signal that promotes the proliferation and suppression of apoptosis.

## MATERIALS AND METHODS

### Patient samples

This study enrolled 238 patients with hepatocellular carcinoma (HCC) from July 2007 to July 2014 from medical center of Chung Ho Memorial Hospital with hepatitis B virus (HBV) and/or hepatitis C virus (HCV) infection and 234 non-HCC patients, of whom 175 were infected with HBV or HCV and 59 were not infected with either virus. Of these patients, 238 had adequate follow-up data for detailed analysis. The study of human subjects was approved by the Institutional Review Board of Kaohsiung Medical University (KMUHIRB-20130052; Kaohsiung, Taiwan).

### Plasmids and cell lines

Full-length *ISX* and *E2F1* cDNA was PCR amplified from a human placenta cDNA library (GIBCO/BRL) were sub cloned into the *pEGFP/C1* or *pCDNA3/HA* vector (CloneTech.) to express the GFP-tagged *ISX* (*E2F1*) protein or the HA-tagged *ISX* (*E2F1*) protein. *ISX* inducible systems in Huh 7 or SK-Hep1 cells were established by lentivirus infection [1]. GFP-*ISX* was subcloned into the *pAS4W.puro* vector (RNAi Core Center, Taiwan) to establish Tet on-inducible GFP-*ISX* expression system. *PGIPZ* was used for *ISX* (*E2F1*) shRNAi construction. The following sequences were used to construct *ISX* shRNAi; shRNAi-1-1(1031-1051 bps): 5′-TGAGCCTGTCCTTCTCCATTG-3′ and shRNAi-2 (1367-1387 bps): 5′-AGCAGGAGAAGATTGGCAACC-3′. The SK-Hep1, Huh 7, Hep G2 and Hep 3B cell lines were sub cultured and maintained according to ATCC protocol. Transfection was performed using the LipofectAMINE transfection kit (GIBCO/BRL).

### Westernblot and immunohistochemical staining analysis

Western blot analysis and immunohistochemical (fluorescence) staining were done as previously described [40, 41]. The primary antibodies used in this study were actin polyclonal antibody (1:5,000, Sigma/ Aldrich), cdc25A, CDK1, c-Myc and cyclin D1 polyclonal antibody (1:1,000, Sigma/Aldrich), E2F1 antibody (1:1,000, Cell Signaling Technology, Beverly MA), GFP monoclonal antibody (1:200, Upstate, Lake Placid, NY), FITC-conjugated anti–rabbit, Rhodamine-conjugated anti-mouse, alkaline phosphatase–conjugated anti-rabbit antibody (1:500, Jackson ImmunoResearch Laboratories, West Grove, PA), and ISX, RB1, Skp2, VPS34, ATG12 and LC3-II rabbit polyclonal antibody (1:200; Santa Cruz). B23, p65, IkBα, Apaf-1, Bcl-2 and Mcl-1monoclonal antibodies (Santa Cruz BioTech. Inc.), Phospho-IκBα, antibodies (1:500, Cell Signaling Tech.). All of experiments repeated at least three times.

### Chromatin immunoprecipitation (ChIP) assays

The chromatin immunoprecipitation (ChIP) assays were analyzed as previous described [40]. All of experiments represents as means ± SD and repeats at least three times. *E2F1* promoter fragment was amplified by PCR with the following primers: primer 1, 5′-TACGCCTGCAACCGTTTAAT-3′; primer 2, 5′-TTTTCCTCCCGGTAGGCTTG-3′.

### Luciferase reporter assays

The expression constructs and two reporter constructs, pSV40-Rluc and pGL3-*E2F1* promoter/Fire luciferase (Promega), were co-transfected with pEGFP/c1-ISX into 2 × 10^5^ Hep 3B cells [41]. All of experiments represents as means ± SD and repeated at least three times. Cells were harvested 16 hours after transfection and relative luciferase activities were measured according to the manufacturer's instructions.

### Electrophoresis mobility shift assay (EMSA)

The DNA binding reaction was performed as described previously [40]. The process is described simply as follows: The nuclear protein extracted from cells (Novagen) and ^32^P-labeled oligonucleotides were incubated in buffer containing 10 mM Tris-HCl, 1 mM EDTA, 100 mM NaCl, 2 mM dithiothreitol (DTT) and 10% glycerol. An 18-mer double-stranded oligo-nucleotide of different promoter elements was used as a probe to interact with nuclear ISX. The antibody against GFP was added 30 min before mixing nuclear protein with oligonucleotides in a super-shift assay. Five micrograms of nuclear protein was incubated with 5000 cpm [^32^P]-labeled oligo-nucleotides, 2 μg poly (dI:dC), and BSA (1 μg/mL) for 30 min at room temperature. The DNA/protein mixtures were separated on a 5% polyacrylamide gel (30:1 bis-acrylamide in 0.5x TBE).

### Real-time polymerase chain reaction (PCR)

The ISX, cyclin *D1*, RB, p53 and E2F1 mRNA expression from hepatoma cells and tumor patients was detected by SYBR Green Quantitative RT-PCR kit (Invitrogen) as previous described [41]. The total RNA was extracted from the tumor mass with Trizol reagent (Invitrogen), and then transcribed into cDNA (Invitrogen) for PCR amplification using an ABI 7900HT Thermocycler. All reactions and data analyses were performed according to the manufacturer's instructions. pEGFP/c1 vector-transfected cells and samples from normal subjects and drug treated patients were analyzed for comparison. All of experiments represents as means±SD and repeated at least three times.

### Flow cytometry analysis of apoptosis and autophagy

Annexin V-FITC/PI Detection Kit was used for the determination of cell apoptosis. Huh 7 cells transfected with vector, ISX and/or E2F1were plated in 6-well plates at the density of 1 × 10^6^ cells/well. After 24 h, cells were treated with Tamoxifen (30mM) for 8 hours and be harvested for analysis. The hepatoma cells were washed twice with ice-cold PBS and re-suspended in 500 μl binding buffer at a concentration of 1 × 10 6 cells/ml. According to the manufacturer's description, cells were incubated in the dark with 5 μl Annexin V-FITC (fluorescein isothiocyanate) and 5 μl PI (propidium iodide) for 30 min at room temperature. Cell autophagy was examined by detecting acidic vesicular organelles (AVO) using according orange (AO) stain. Cells were stained with 1 mg/mL AO for 30 min and collected in PBS. The rate of apoptosis and autophagy were immediately analyzed by FACSCalibur flow cytometer. Analysis was carried out by triplicate determination on at least three separate experiments.

### Anchorage-independent growth assays

Cells (10^4^ or 5 × 10^3^) in 1-mL culture medium were mixed with an equal volume of 0.6% of top agar and plated onto 60-mm dishes with 0.5% bottom agar [41] Plates were incubated at 37°C for 2 weeks. Colonies were visualized by staining with 0.05% crystal violet acetate and colonies larger than 0.5 mm were counted. The culture medium refreshed every 3 days. All of experiments represents as means ± SD and repeated at least three times.

### Tumorigenic assay of ISX-E2F1 axis signaling in nude mice assay

Female BALB/c nu/nu mice were obtained from the National Laboratory of Animal Breeding and Research Center (Taipei, Taiwan). Nude mice were inoculated (s.c. injection) with 10^6^ vector- or ISX (E2F1) shRNAi -transfected cells individually on either side of the back (n = 10 mice/group) [41]. Tumor size was measured with a caliper once or twice a week. The tumor volume was estimated according to the formula: volume (cm^3^) = 1/2(LXW^2^), where L and W are the length and width of the tumor, respectively.

### Statistical analysis

The quantitative variables are presented as the mean ± SD. The significance of differences was determined using a two-sample t-test. *Pearson's* and *Spearman rank* correlational analysis was used to examine the relationship between the expression levels of *ISX, E2F1*, and *RB1*. Statistical analysis of categorical variables was performed using chi-squared analysis, one-way analysis of variance (ANOVA), and Fisher's exact analysis. Differences with a *p* value <0.05 were considered to be significant.

## SUPPLEMENTARY FIGURE


