# Supplementary material for: Intestine-specific homeobox (ISX) upregulates E2F1 expression and related oncogenic activities in HCC
Source: Oncotarget. 2016 May 9;7(24):36924–39. doi: 10.18632/oncotarget.9228 (PMC5095049; doi:10.18632/oncotarget.9228)
Supplement: Supplementary file 1 [file oncotarget-07-36924-s001.pdf]

Intestine-specific homeobox (ISX) upregulates E2F1 expression and related oncogenic activities in HCC

SUPPLEMENTARY FIGURE

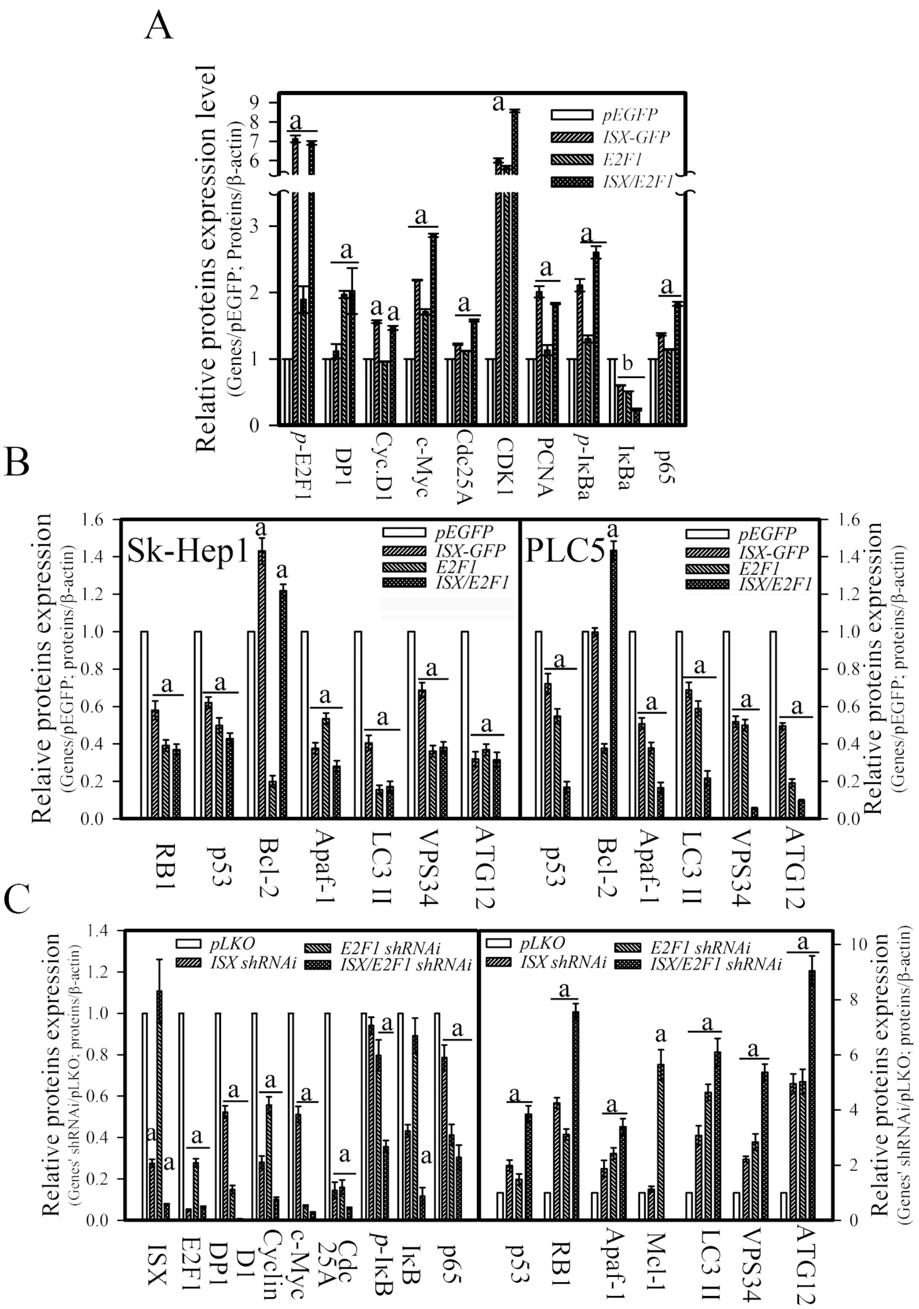

Supplementary Figure S1: Relative expression levels of proteins in cells induced by forced expression vectors ISX and/or E2F1 and knockdown vectors ISX and/or E2F1. A. Relative expression levels of protein in SK-Hep1 cells were calculated from the intensities of the respective bands shown in Figure 3D. **B.** Relative expression levels of protein in SK-Hep1 and PLC5 cells were calculated from the intensities of the respective bands shown in Figure 3E. **C.** Relative expression levels of protein in SK-Hep1 and Huh 7 cells were calculated from the intensities of respective bands shown in Figure 3F. All results are shown as means ± S.D. ($N = 3$), a, $p < 0.001$.
